# Raman Spectroscopic Authentication of Rebaudioside M: Discriminating Natural, Fermentation-Derived, and Enzymatically Bioconverted Stevia Sweeteners

**DOI:** 10.3390/foods15111994

**Published:** 2026-06-03

**Authors:** Giuseppe Pezzotti, Akihiro Miyamoto, Takashi Yamashita, Isao Fujita, Akihiro Maeno, Wenliang Zhu, Manabu Nakagawa, Takuya Kobayashi

**Affiliations:** 1 Biomedical Engineering Center, Kansai Medical University, 1-9-11 Shin-Machi, Hirakata 573-1191, Japan; 2 International Center for Biomedical Industrial Promotion, Kansai Medical University, 1-9-11 Shin-Machi, Hirakata 573-1191, Japan; 3 Department of Immunology, Graduate School of Medical Science, Kyoto Prefectural University of Medicine, Kamigyo-Ku, 465 Kajii-Cho, Kyoto 602-8566, Japan; 4 Department of Orthopedic Surgery, Tokyo Medical University, 6-7-1 Nishi-Shinjuku, Shinjuku-Ku 160-0023, Japan; 5 Department of Molecular Science and Nanosystems, Ca’ Foscari University of Venice, Via Torino 155, 30172 Venice, Italy; 6 Biomarker Disease Laboratory, IRCCS San Camillo Hospital, Via Alberoni 70, 30126 Venice Lido, Italy; 7 Department of Orthopaedic Surgery, Graduate School of Medicine, Mie University, 2-174 Edobashi, Tsu 514-8507, Japan; 8 Morita Kagaku Kogyo Co., Ltd., 1-19-18 Inadauemachi, Higashi Osaka 577-0002, Japan; 9 Ceramic Physics Laboratory, Kyoto Institute of Technology, Sakyo-Ku, Matsugasaki, Kyoto 606-8585, Japan; 10 Department of Medical Chemistry, Kansai Medical University, 2-5-1 Shinmachi, Hirakata 573-1010, Japan; maeno.aki@kmu.ac.jp (A.M.);

**Keywords:** Rebaudioside M, Raman spectroscopy, authentication

## Abstract

Rebaudioside M (Reb M) is a high-value steviol glycoside responsible for the most desirable sensorial profile among stevia-derived sweeteners, owing to its intense sweetness and near absence of bitter aftertaste. However, its extremely low natural abundance in *Stevia rebaudiana* leaves has driven the development of alternative production strategies, including microbial fermentation and enzyme-assisted bioconversion. In this work, Raman spectroscopy is employed as a rapid, non-destructive, and label-free analytical tool to discriminate Reb M obtained from three distinct sources: (i) naturally occurring leaf extracts, (ii) fermentation-derived products, and (iii) enzymatically bioconverted products. Distinct vibrational fingerprints are identified that reflect differences in glycosylation patterns, residual steviol glycoside populations, matrix components, and process-related byproducts. The results demonstrate that Raman spectroscopy enables prompt authentication of Reb M origin and provides a powerful platform for real-time quality control. Importantly, the technique allows near-zero-cost screening, thus offering a decisive advantage over conventional chromatographic methods. These findings highlight Raman spectroscopy as a key method enabling a swift procedure for ensuring transparency, safety, and consistency in next-generation Stevia sweeteners.

## 1. Introduction

The global demand for non-caloric/low-caloric, natural sweeteners has increased dramatically in response to rising concerns over obesity, diabetes, and other metabolic disorders [1,2]. Among plants that contain sweeteners, *Stevia rebaudiana* Bertoni has attracted exceptional attention due to its content of steviol glycosides, a family of diterpenoid compounds characterized by sweetness intensities up to several hundred times that of sucrose [3]. However, not all steviol glycosides contribute equally to sensorial quality. Early commercial products were dominated by stevioside and rebaudioside A (Reb A), which, despite their sweetness, are often associated with bitterness and lingering aftertaste [4]. Within the family of steviol glycosides, rebaudioside M (Reb M) has emerged as a superior sweetening compound because of its clean, sugar-like taste and minimal off-flavors [5]. Structurally, Reb M differs from earlier steviol glycosides by a higher degree of glycosylation, which modulates receptor interactions on the human taste bud cells and suppresses bitter perception [6]. Nevertheless, Reb M is present in *Stevia* leaves at extremely low concentrations (typically less than 0.1% of total steviol glycosides) making direct extraction economically inefficient and environmentally burdensome [7]. This intrinsic limitation of the natural source has motivated the development of alternative production routes, which are presently flourishing all around the world.

Microbial fermentation represents the first major technological response to the scarcity of Reb M. In this approach, genetically engineered microorganisms, such as yeast or bacteria, are designed to biosynthesize steviol or steviol glycosides through reconstructed metabolic pathways [8]. Fermentation enables scalable production and reduces dependency on agricultural variables such as climate, soil quality, and harvest yield. However, fermentation-derived Reb M often coexists with structurally related intermediates, residual sugars, or fermentation byproducts that may affect purity, sensorial performance, or safety, if not rigorously controlled [9].

Enzyme-assisted bioconversion has subsequently emerged as a complementary and, in many cases, superior strategy. This technology typically starts from more abundant steviol glycosides (e.g., Reb A or stevioside) extracted from *Stevia* leaves and employs specific glycosyltransferases or whole-cell biocatalysts to selectively add glucose moieties, converting them into Reb M [10,11]. Bioconversion offers higher structural fidelity, improved yield, and a molecular profile closer to that of naturally occurring Reb M, while maintaining a favorable regulatory status as a nature-identical process [12]. Understanding and distinguishing the subtle molecular differences arising from these production routes is therefore essential for both manufacturers and regulatory authorities.

Analytical characterization of Reb M has traditionally relied on chromatographic techniques such as high-performance liquid chromatography and liquid chromatography–mass spectrometry [13]. While powerful, these methods require extensive sample preparation, trained personnel, and significant operational costs. In contrast, Raman spectroscopy provides a rapid, non-invasive alternative that directly probes molecular vibrations, offering a chemically specific fingerprint in nutrition science [14]. Raman spectroscopy has already demonstrated strong potential in food science for authenticity assessment, detection of adulterants, and monitoring of biochemical transformations [15,16].

In the context of stevia sweeteners, Raman spectroscopy is uniquely positioned to differentiate Reb M produced by natural extraction, fermentation, and bioconversion, by capturing variations in glycosidic linkages, diterpene backbone vibrations, and accompanying matrix components [17]. Such variations, which directly link to the sweetness/bitterness paradigmatic characteristic of stevia products, allow the classification of product quality with a completely non-destructive, fast and low-cost procedure. Moreover, from the consumers’ perspective, the Raman technique enables immediate detection of undeclared artificial sweeteners, such as aspartame, acesulfame K, or sucralose, through their distinct and easily recognizable Raman signatures [18]. In summary, this spectroscopic capability offers a decisive advantage for routine quality control, counterfeit prevention, and regulatory compliance, all at near-zero cost once instrumentation is in place.

Building upon our previously published research on elementary rebaudioside molecules [17], the present study leverages Raman spectroscopy to systematically compare commercially available Reb M obtained from leaf extraction, microbial fermentation, and enzymatic bioconversion. The main motivation for this work resided in the need to ascertain the quality of commercial stevia products, which nowadays have become very numerous but yet lack an appropriate and scientifically validated evaluation method. By rigorously correlating spectral features with production pathways, we demonstrate here the feasibility of Raman-based authentication and quality assessment as a robust tool for next-generation sweetener technologies. The implementation of a Raman technology approach in the commercialization of new stevia products not only will support their faster development, but will also provide a clear path for their quality control before market release.

## 2. Experimental Procedures

### 2.1. Samples Obtained from Different Manufacturing Procedures

Five Reb M products, which were commercially available as natural products, were analyzed from different lots, as follows: one sample from Morita Kagaku Kogyo, Osaka, Japan (two sample lots labeled as A1-1 and A1-2), two samples from the USA (labeled as A2-1/A2-2 and A5-1/A5-2/A5-3) and two samples from China (labeled as A3-1/A3-2 and A4-1/A4-2/A4-3). Three Reb M commercially available samples produced by microbial fermentation were analyzed in different lots, all from the USA (labeled as B1-1/B2-2, B2-1/B2-2, and B3-1/B3-2/B3-3). Four enzyme-assisted bioconverted commercially available samples in different lots: two from the UK (labeled as C1-1 and C4-1/C4-2/C4-3), and two samples from the USA (labeled as C2-1/C2-2 and C3-1/C3-2/C3-3).

### 2.2. Raman Spectroscopy

The Raman spectral library of the elementary diterpene glycoside molecules and the spectra of commercially available products were all compiled by means of a high spectrally resolved Raman device equipped with a triple-monochromator (T-64000; Jobin-Ivon/Horiba Group, Kyoto, Japan) and a nitrogen-cooled charge-coupled device detector (CCD-3500V, Jobin-Ivon/Horiba Group, Kyoto, Japan). The excitation source was the 514 nm line of an Ar-ion laser operating with a nominal power of 200 mW. The spectral resolution was better than 0.5 cm^−1^. Series of 10 spectra were collected (with a 50× optical lens) at different locations of each sample and then averaged to obtain a representative spectrum per each compound or product. The collected Raman spectra were subjected to polynomial baseline subtraction and deconvolution into a series of Gaussian–Lorentzian band components. The baseline subtraction procedure was performed using options available in commercial software (LabSpec 4.02, Horiba/Jobin-Yvon, Kyoto, Japan) with fixed criteria for all collected spectra. All spectra were analyzed after intensity normalization to the strongest signal in the collected spectral interval. Spectral deconvolution was carried out by applying a machine-learning algorithm described in a previously published paper [19], and implemented with spectral data collected on pure rebaudioside molecules [17].

### 2.3. Statistical Analyses

Data are presented as a mean value ± standard deviation of five independent measurements on each lot of each product (*n* = 5). Statistical differences were analyzed by means of unpaired Student’s *t*-test. A value *p* < 0.05 was considered as statistically significant and labeled with two asterisks.

## 3. Experimental Results

**Figure 1:**
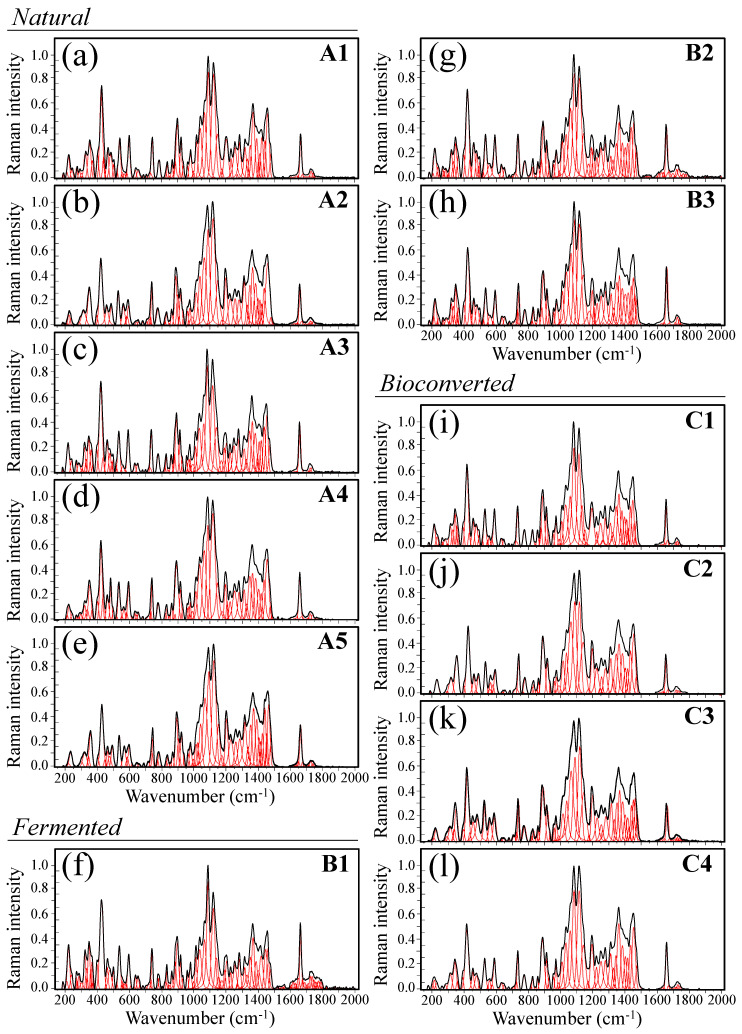
Raman spectra of Rebaudioside M prepared by different procedures. Average and deconvoluted Raman spectra collected over different lots of Reb M products extracted from natural Stevia leaves (5 different products, A1~A5, referred to as “natural”) (**a**–**e**), manufactured by bacterial fermentation (3 different products, B1~B3, referred to as “fermented”) (**f**–**h**), and enzymatically bioconverted (4 different products, C1~C4, referred to as “bioconverted”) (**i**–**l**) (cf. labels in inset).

Figure 1a–l show average and deconvoluted Raman spectra collected over different lots of all Reb M products investigated. The figures comprise spectra from products extracted from natural stevia leaves (5 different products, A1~A5, referred to as “natural”), products manufactured by bacterial fermentation (3 different products, B1~B3, referred to as “fermented”), and products enzymatically bioconverted (4 different products, C1~C4, referred to as “bioconverted”) (cf. labels in inset). All spectra, as expected, showed strong similarities, with strong signals being located within the wavenumber interval 1085~1120 cm^−1^. These signals relate to C–O or C–O–C stretching vibrations. Despite such strong similarity at a glance, a number of subtle but important differences could also be resolved. Some main spectral features are pointed out in the following, while a more complete description of key spectral differences for extracting information on the overall product quality will be given in the forthcoming discussion in Section 4.

Relatively easy to spot was a distinct and reproducible enhancement of Raman intensity in the low-wavenumber region (200~250 cm^−1^), which was systematically observed in fermentation-derived samples compared with both leaf-extracted and enzymatically bioconverted products. Sulfur-related signals, typical of this spectral zone, should be absent in the absence of contamination. In microbial fermentation systems, sulfur is indeed present in cysteine and methionine amino acid residues. However, the purification pipelines used for high-value steviol glycosides should be able to remove proteins, peptides, and low-molecular-weight metabolites efficiently. An alternative explanation may invoke vibrational modes that are generally associated with lattice phonons, intermolecular collective motions, and skeletal torsional modes involving heavy-atom frameworks, rather than with localized intramolecular stretching vibrations typical of higher wavenumbers. In favor of this latter spectral interpretation is the fact that such signals are also present in highly purified Reb M (cf. later statements in this paragraph), although the pure molecule is predominantly characterized by intramolecular glycosidic and C–C/C–O vibrational contributions occurring above 400 cm^−1^. While the amplified low-wavenumber features seen in fermented materials might also be affected by bacterial contamination in overlap with supramolecular or solid-state effects, the most plausible origin of such intensity enhancement may reside in the presence of microcrystalline domains with altered packing symmetry or increased lattice disorder. These supramolecular characteristics can enhance low-frequency phonon density of states. Fermentation processes may also introduce residual inorganic species (e.g., trace metal ions from culture media), fermentation-derived secondary metabolites, or structurally related steviol glycosides that modify intermolecular interactions and crystal cohesion, thereby increasing lattice-mode Raman activity. In contrast, products obtained via direct leaf extraction undergo plant-specific biosynthetic control and purification pathways that tend to preserve a more homogeneous glycoside matrix. Enzymatic bioconversion, being a targeted and substrate-specific transformation, appears to generate a composition closer to the parent crystalline Reb M phase, resulting in weaker collective vibrational signatures in this region. The enhanced 200~250 cm^−1^ signals in fermented products therefore are interpreted here as mainly originating from process-dependent differences in solid-state organization, residual matrix components, and lattice dynamics, providing spectroscopic evidence of distinct supramolecular architectures. This low-wavenumber window emerges as a sensitive marker of manufacturing-induced structural heterogeneity and offers additional discriminatory power for assessing origin and quality in steviol glycoside sweeteners. Additional discussions on the structural order of differently manufactured samples will be given in Section 4.1.

The fermented product B1 also exhibited up to 38~40% more pronounced intensity for the amide I band (1650~1665 cm^−1^) relative to both natural and enzymatically bioconverted samples. As Reb M itself should contain a quite low, although not completely zero, amount of nitrogenous functionalities, this feature is mainly attributed to trace fermentation-derived proteinaceous or peptide residues, likely originating from microbial biomass or enzymatic catalysts used during production. The strong sensitivity of the amide I band arises from the high Raman cross-section of its amide carbonyl vibrations with respect to any compositional variation.

**Figure 2:**
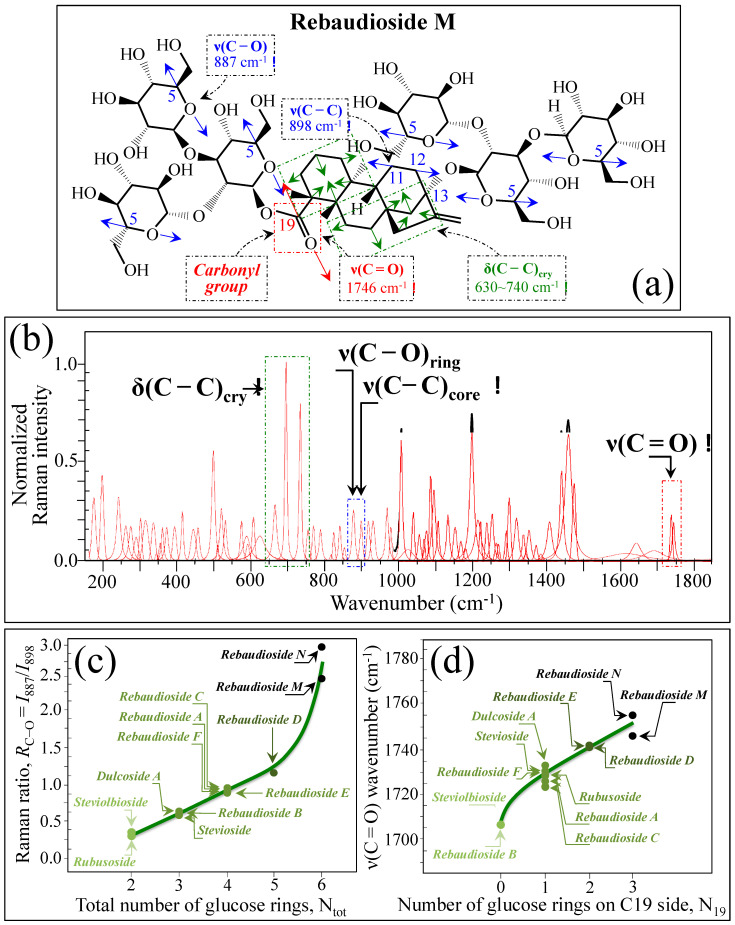
Definition of molecular Raman markers. (**a**) Molecular structure and (**b**) Raman spectrum of pure Reb M, with its aglycone core surrounded by six glucose rings, three per each C19 and C13 side of the molecule; broken box in (**b**) represents characteristic signals of interest (cf. label inset), whose vibrational origins are shown in (**a**). In (**c**,**d**), calibration curves given [17], which will be employed in Section 4.2 to evaluate both glycosidic completeness and sensorial performance of commercial stevia products.

Figure 2a,b shows the molecular structure and the Raman spectrum of pure Reb M, with its aglycone core surrounded by six glucose rings, three per each C19 and C13 side of the molecule [17]. In these figures, Raman vibrational modes of interest are explicitly emphasized together with their expected wavenumbers (cf. labels in inset in (a) and bands comprised within broken square intervals in (b)). In particular, signals from C5–O stretching in glucose rings (at ~887 cm^−1^) and from C11–C12 stretching in the aglycone core (at ~898 cm^−1^), and C=O carbonyl stretching at ~1746 cm^−1^ contain important information about the molecular structure. In a previous paper [17], we examined the Raman spectra of a full library of highly pure rebaudioside molecules and established Raman spectroscopic parameters capable to link the observed spectrum to the intrinsic structure of the molecule. Two relevant Raman ratios could be retrieved. One is the areal ratio, R_C–O_ = *I*_887_/*I*_898_ between the area subtended by the 887 cm^−1^ signal from C5–O stretching in glucose rings and that of the 887 cm^−1^ signal from C11–C12 stretching in the aglycone core. This ratio, as shown in the calibration curve of Figure 2c, is propaedeutic of the total number of rings, N_tot_, present in the spectroscopically analyzed rebaudioside molecule. The second structural marker is the wavenumber at maximum of the carbonyl stretching, ν_C=O_, which could be calibrated to retrieve the number of rings on the C19 side, N_19_, as shown in Figure 2d. The calibration curves, which are given in Figure 2c,d, will be employed in the forthcoming Section 4.2 to evaluate both glycosidic completeness and sensorial performance of commercial stevia products. An important feature in the context of C=O stretching is the complete absence of signals above 1750 cm^−1^ in the spectrum of pure Reb M and, in general, in spectra of any pure rebaudioside molecule (cf. Figure 2d). The presence of a family of signals at above 1750 cm^−1^ in the spectrum of several commercial stevia products (especially the fermented ones; cf. Figure 1f,g) represents an important marker of the quality of the manufacturing process, since such signals are unequivocally related to the chemical integrity of the product. Screening the relative intensities of such signals enables us to track back the presence of trace processing-derived diterpenoid lactones and oxidized steviol fragments. This point will be discussed in detail in the forthcoming Section 4.3.

Finally, the sharp triplet at 665/694/730 cm^−1^ in the pure Reb M compound represents another important feature in the spectrum of pure Reb M (cf. δ(C–C)_cry_ in Figure 2b). These signals, which can be assigned to C–C skeletal and ring deformation modes in the steviol aglycone core, are highly sensitive to molecular packing and crystallinity. In particular, an index of molecular crystallinity, R_cry_ = (I_split_)_com_/(I_665_ + I_694_ + I_730_)_p_, can be assumed as the ratio between the cumulative area subtended by the deconvoluted bands in the interval, 625~775 cm^−1^ in the spectrum of commercial products, (I_split_)_com_, and the area subtended by the 665/694/730 cm^−1^ triplet in the pure Reb M sample, (I_665_ + I_694_ + I_730_)_p_ (cf. Figure 2b). We will employ this notion in Section 4.1 in order to establish a Raman parameter describing molecular packing and crystallinity as a marker of homogeneity in commercial stevia products.

## 4. Discussion

### 4.1. Heterogeneity and Molecular Crystallinity Markers

**Figure 3:**
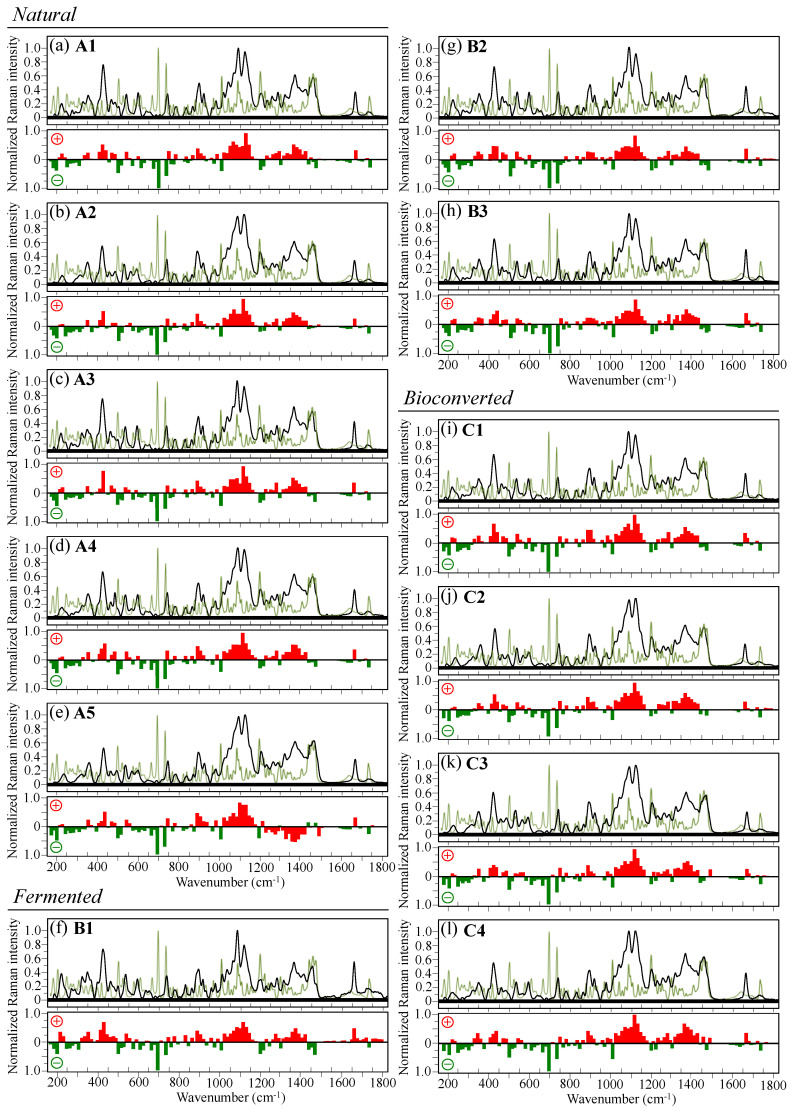
Comparison between commercial products and standard molecule. Spectral comparison and difference spectra computed for all commercial products examined in this study (cf. labels in inset) with respect to the spectrum of pure Reb M in Figure 2b; natural products: (**a**–**e**), fermented products (**f**–**h**), and bioconverted products (**i**–**l**).

**Figure 4:**
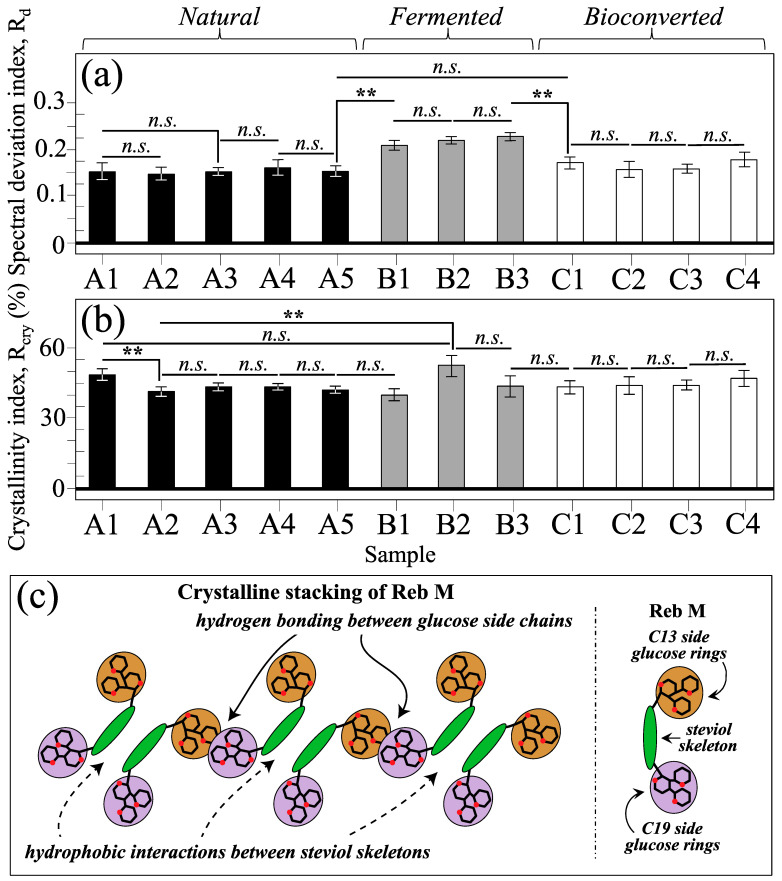
Comparison of molecular markers in commercial products. (**a**) Spectral deviation index, R_D_, as computed as an average over the entire wavenumber interval investigated of the absolute-value of the Raman intensity difference between the normalized spectrum of each commercial product and that of the highly pure Reb M (cf. Figure 3). The standard deviations shown refer to different lots within each product investigated. In (**b**), a plot is given with the index of molecular crystallinity, R_cry_ = (I_split_)_com_/(I_665_ + I_694_ + I_730_)_p_, assumed as the ratio between the cumulative area subtended by the deconvoluted bands in the interval, 625~775 cm^−1^ in the spectrum of commercial products, (I_split_)_com_ (cf. Figure 5), and the area subtended by the 665/694/730 cm^−1^ triplet in the pure Reb M sample, (I_665_ + I_694_ + I_730_)_p_ (cf. Figure 2b). A schematic draft of crystallinity stacking in pure Reb M is shown in (**c**), as elaborated from Ref. [20]. Statistically non-significant difference were labeled as *n.s.*, while a value *p* < 0.05 was considered as statistically significant and labeled with two asterisks.

Comparison of the Raman spectra of commercially available Reb M products with that of the highly pure Reb M reference revealed pronounced and systematic differences that could be explained by crystalline formulation in addition to differences in molecular structure. Figure 3a–l shows spectral comparisons and difference-spectra plots computed with respect to the spectrum of pure Reb M (in Figure 2b), as built up for all commercial products examined (cf. labels in inset). From such difference spectra, a spectral deviation index, R_D_, could be extracted as the absolute value of the average intensity difference between the normalized spectrum of the commercial product and that of pure Reb M, as computed over the entire wavenumber interval studied. This computed spectroscopic parameter is plotted in Figure 4a with its related standard deviation as computed among different lots within each investigated product. The plot in Figure 4a shows that all commercial products examined in this study were highly heterogeneous, since significantly diverging from pure Reb M. While statistically non-significant differences were found among spectra belonging to samples produced by the same manufacturing procedure, all samples produced by fermentation were statistically more inhomogeneous than both natural and bioconverted ones (cf. results of statistical analysis in inset to Figure 4a). A quantitative assessment of the level of inhomogeneity of the studied Reb M samples (total number of 28 investigated samples), with respect to both different fabrication procedures and heterogeneity across different batches of the same product, was obtained by applying a principal component analysis (PCA) to the recorded Raman spectra of all the investigated samples. This statistical analysis is provided in Figure S1 of Supplementary Information. The PCA, performed on the entire Raman spectrum, confirmed that the highest level of inhomogeneity stemmed among microbially fermented samples.

Spectral comparison and difference spectra computed for all commercial products examined in this study (cf. labels in inset) with respect to the spectrum of pure Reb M in Figure 2b).

**Figure 5:**
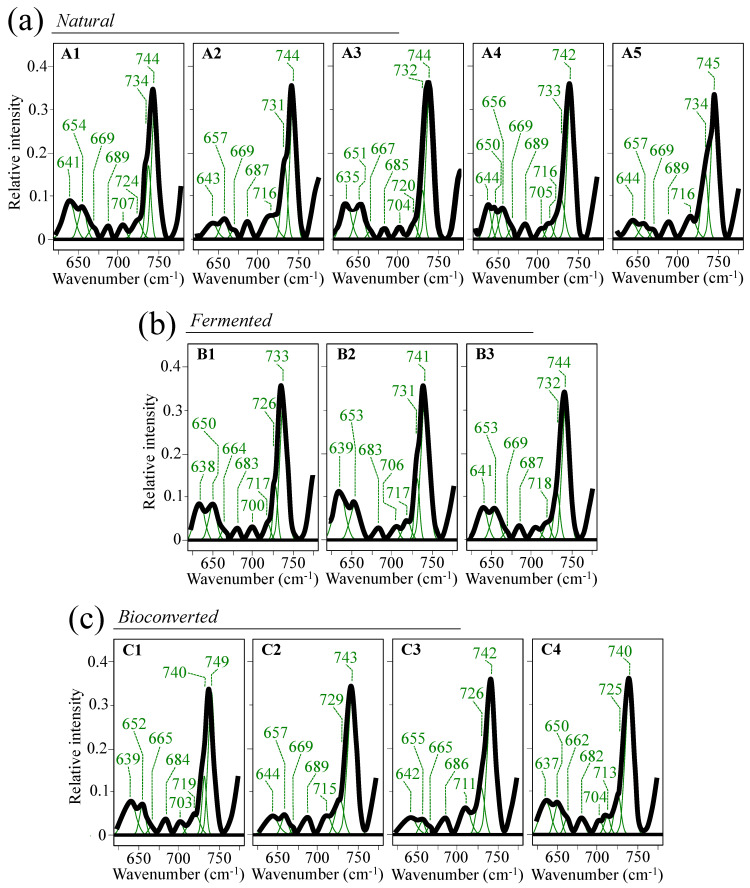
Raman analysis in the 625~775 cm^−1^ wavenumber interval; the 625~775 cm^−1^ wavenumber interval as extracted from the spectra in Figure 3 after enlargement and deconvolution reveals a split ensemble of 6~8 weak, broad, and shifted signals in commercial stevia products to replace the sharp 665/694/730 cm^−1^ observed in the pure Reb M compound (cf. wavenumbers in cm^−1^ reported in inset). Spectra for natural, fermented, and bioconverted samples are given in (**a**), (**b**), and (**c**), respectively.

The most striking difference between spectra collected on commercial products and the spectrum of pure Reb M was recorded in the wavenumber interval 625~775 cm^−1^ (cf. Figure 2b and Figure 3). The pure Reb M reference sample exhibited its strongest band at ~694 cm^−1^, with sharp neighboring bands at 665 and 730 cm^−1^ on its lower and higher wavenumber sides, respectively (cf. broken-line box in Figure 2b). However, these three bands either completely disappeared or shifted, while becoming very weak and broad in all commercial samples regardless of whether they were obtained by natural extraction, fermentation, or enzymatic bioconversion (cf. Figure 3). Looking into more detail in this spectral region, the strong triplet 665/694/730 cm^−1^ in the pure Reb M compound transformed into a split ensemble of 6~8 much weaker, broad and shifted signals at wavenumbers in the interval 625~750 cm^−1^. These spectral features are better shown in Figure 5, in which the 625~775 cm^−1^ interval is extracted from the spectra in Figure 3, enlarged, and deconvoluted. The triplet 665/694/730 cm^−1^ in the pure Reb M compound arises from C–C skeletal and ring deformation modes of the steviol aglycone core and is highly sensitive to molecular packing and crystallinity. The present spectral observations suggest that the reference (pure) Reb M sample is predominantly crystalline, whereas commercial products are largely amorphous or structurally disordered. Molecular crystallinity in Reb M should be understood not as an intrinsic property of the sugar molecule itself, but as a manifestation of a long- to intermediate-range order arising from the collective packing of identical molecules in the solid state, in close analogy to well-established systems such as crystalline versus amorphous lactose [20]. In a highly ordered Reb M phase, chemically identical molecules adopt a narrow conformational distribution and assemble into a periodic supramolecular lattice stabilized primarily by an extensive and cooperative hydrogen-bonding network involving the multiple hydroxy groups of the glycosyl moieties [21]. Intermolecular glucose–glucose hydrogen bonds, particularly those involving equatorial OH groups and hydroxymethyl functionalities, are expected to form directional “zipper-like” motifs that propagate from one-dimensional chains into two- and three-dimensional lattices, while the rigid steviol aglycone cores act as hydrophobic spacers that promote regular packing and reduce conformational entropy. Additional ordering arises from the alignment of glycosidic C–O–C linkages, whose torsional angles become narrowly distributed in the crystalline state, enabling vibrational coupling between adjacent molecules. In the above context, Raman spectroscopy is exceptionally sensitive to crystallinity, as periodic packing enhances vibrational coherence, narrows bandwidths, and selectively amplifies collective skeletal modes. The intense and sharp Raman triplet at 665/694/730 cm^−1^, only observed in highly pure and well-ordered Reb M (cf. Figure 2b and Figure 3), can therefore be rationalized as a set of molecular vibrations involving deformations and affected by glycosidic linkages constrained within a highly ordered lattice; when this order is disrupted, as in amorphous or poorly crystalline commercial products, the same modes become broadened, weakened, or effectively extinguished. Importantly, the weakness of this triplet in Reb M obtained via natural extraction, fermentation, or enzymatic biotransformation does not imply chemical impurity, but rather reflects the predominance of amorphous domains, conformational heterogeneity, residual water or solvents, and rapid precipitation or drying processes that kinetically trap disorder. Thus, the 665/694/730 cm^−1^ triplet should be regarded as a solid-state order marker rather than a compositional one, highlighting Raman spectroscopy as a powerful, complementary tool to chromatographic methods for probing crystallinity, processing history, and supramolecular organization in complex glycosylated sweeteners. Note that, from a vibrational viewpoint, a shift towards lower wavenumbers could be interpreted as the consequence of an increased conformational freedom of the molecular framework, while splitting could be a marker for the presence of two non-equivalent local environments. As defined in Section 3, an index of molecular crystallinity, R_cry_ = (I_split_)_com_/(I_665_ + I_694_ + I_730_)_p_, was then assumed as the ratio in relative intensity between the cumulative area subtended by the series of deconvoluted bands in the interval, 625~775 cm^−1^ in the spectrum of commercial products, (I_split_)_com_ (cf. Figure 5), and the area subtended by the 665/694/730 cm^−1^ triplet in the pure Reb M sample, (I_665_ + I_694_ + I_730_)_p_ (cf. Figure 2b). A plot of crystallinity index is given in Figure 4b. As can be seen, none of the commercial products reached a percent crystallinity level higher than ~50% of that of the pure molecule with the exception of the fermented sample B2 (R_cry_ = 52.9%), while the natural sample A1 was the second highest with a crystallinity value slightly below 50%. This means that all commercial samples were in a significantly disordered molecular state, with no long-range translational order. In Figure 4c, a schematic draft is shown for a multidimensional “zipper-like” highly crystalline Reb M lattice (imagining it according to Ref. [21]).

In support of the above order/disorder interpretation, all commercial samples displayed their most intense bands in the 1085~1120 cm^−1^ region. This spectral zone was clearly weaker in the spectrum of the pure Reb M reference (cf. difference spectra in Figure 3). Signals comprised in the wavenumber interval 1085~1120 cm^−1^ are characteristic of C–O and C–O–C stretching vibrations of carbohydrate moieties and are expected to be particularly intense for residual sugars, oligosaccharides, or carbohydrate-based excipients, namely, compounds commonly present at relatively low levels in commercial Reb M as a consequence of preparation procedures. Owing to the large Raman cross-sections of these modes, even minor carbohydrate impurities or formulation components can dominate the spectra. The consistency of these features across all products from different production routes indicates that post-production processing, including purification, drying, hydration state, and excipient addition, governs the observed Raman signatures. These procedures are the main contributors of both sample heterogeneity and amorphization, as characterized by the above-defined spectral deviation index, R_D_, and molecular crystallinity index, R_cry_, respectively.

Overall, the present results vividly demonstrate that Raman spectroscopy is highly sensitive to the solid-state form and compositional matrix of Reb M and that spectral differences between highly pure and commercial materials should be interpreted as evidence of the overall variations in structural homogeneity and molecular crystallinity.

### 4.2. Raman Assessments of Glycosidic Completeness and Sensorial Performance

According to a nowadays well-established knowledge [22,23,24] bitter and sweet taste perception arises from ligand-specific activation of distinct G-protein-coupled receptors, primarily the bitter taste receptors (TAS2Rs) and the sweet taste receptor heterodimer (T1R2/T1R3), with molecular features such as carbonyl functionality, hydrophobic surface exposure, molecular size, and substitution pattern jointly determining receptor selectivity and signaling kinetics. Carbonyl groups contribute to bitterness not intrinsically, but by acting as hydrogen-bond acceptors and dipolar anchoring points that facilitate binding of moderately hydrophobic, compact molecules to TAS2R binding pockets, particularly when the carbonyl is exposed and properly oriented within a largely nonpolar scaffold [25,26,27]. Despite fluctuations in the perception of bitterness among different individuals due to genetic factors [28], the steviol aglycone core, in combination with the specific molecular arrangement of D-glucose rings on both its C19 and C13 sides, is known to be a key-player in producing bitter aftertaste upon interacting with bitter taste receptors [6,29,30]. From a molecular arrangement viewpoint, both spatial positioning and interactions among different functional groups in diterpene molecules greatly influence the overall three-dimensional structure. The spatial hindrance caused by adjacent atoms or groups (usually referred to as steric effect) should clearly impact on binding of the glycoside to taste receptors. In rebaudiosides, despite the presence of a carbonyl group at C19, the overall taste is predominantly sweet because their large molecular size, extensive glycosylation, and high polarity sterically shield the hydrophobic diterpene core and carbonyl functionalities from productive TAS2R interactions, while strongly favoring multivalent hydrogen bonding with the sweet receptor T1R2/T1R3 [4,6,31,32,33]. Despite this, stevia products might exhibit a characteristic lingering aftertaste due to slow dissociation kinetics from the sweet receptor, adsorption to oral surfaces, and delayed, weak activation of certain bitter receptors that becomes perceptible as the dominant sweet signal decays [34,35,36]. Structural variations among steviol glycosides demonstrate that glycosylation at the C13 position is the principal determinant of bitterness suppression and aftertaste quality, as C13-linked sugar chains directly shield receptor-facing hydrophobic regions and modulate receptor residence time, whereas C19 substitution primarily affects solubility and sweetness intensity with only secondary influence on bitterness [37,38,39]. Consequently, increased bulk, branching, or ring number at C13 (as in rebaudioside M, compared with stevioside or rebaudioside A) correlates with reduced bitter aftertaste and a cleaner temporal sweetness profile, underscoring that taste perception is governed by the integrated spatial, kinetic, and receptor-selective context of functional groups rather than their mere presence [6,27,38]. The above notions justify the findings that pure Reb M, with more glucose residues on both C13 and C19 sides, possesses higher maximum sweetness and a faster sweetness perception rate as compared to any other rebaudioside [29]. As additional information, phenomenological data systematically collected by Tian et al. [29] revealed four key adsorption/desorption characteristics of taste receptors, as follows:

(a) Fewer glucosyl groups on C19 result in shorter time for initial stimulation and longer perception of bitterness.
(b) More glucosyl groups on C13 give a faster increase and a stronger intensity of sweetness.
(c) A lower ratio between C13/C19 glucosyl groups leads to a faster sweetness peak perception, while not affecting the bitter taste.
(d) Higher numbers at C19 position lead to a quicker decay of sweetness.

**Figure 6:**
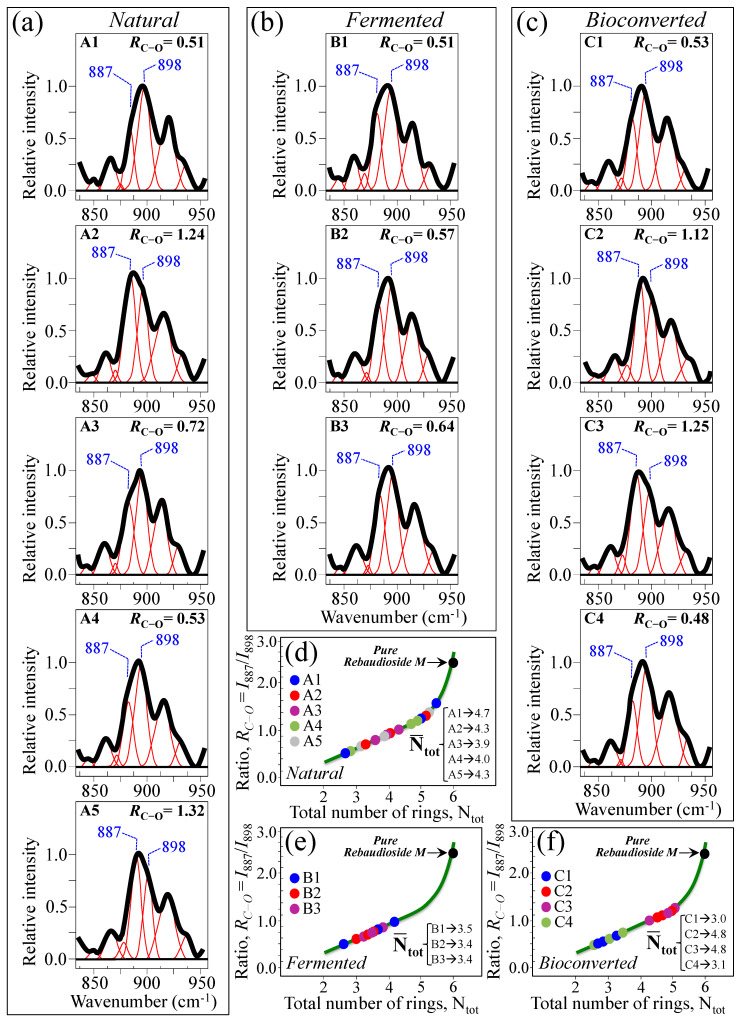
Raman analysis in the 850~950 cm^−1^ wavenumber interval; enlarged and deconvoluted Raman regions in the spectral interval 825~950 cm^−1^ for (**a**) natural (A1~A5), (**b**) fermented (B1~B3), and (**c**) bioconverted (C1~C4) samples, respectively (cf. labels in inset). From this spectral region, the spectroscopic areal ratio between bands at 887 and 898 cm^−1^ was computed in order to retrieve the total number of rings for each commercial product. The calibration plot in Figure 2c is replotted in (**d**–**f**) with adding *R*_C–O_ data collected on different lots of natural, fermented and bioconverted samples, respectively.

**Figure 7:**
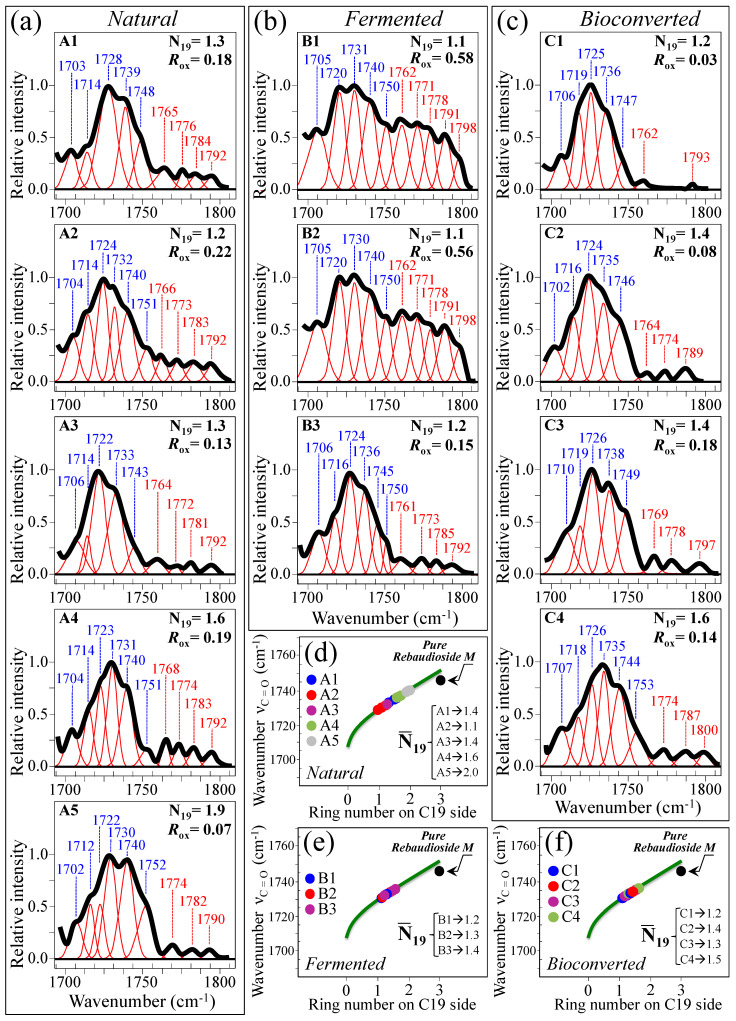
Raman analysis in the 1700~1800 cm^−1^ wavenumber interval; enlarged and deconvoluted Raman regions in the spectral interval 1700~1800 cm^−1^ for (**a**) natural (A1~A5), (**b**) fermented (B1~B3), and (**c**) bioconverted (C1~C4) samples, respectively (cf. labels in inset). From this spectral region, the wavenumbers ν_C=O_ of Raman sub-bands were retrieved in order to compute the number of rings on C19 side for each commercial product from the calibration curve given in Figure 2c and replotted in (**d**–**f**) with adding data collected on different lots of natural, fermented and bioconverted samples, respectively. Wavenumbers of spectral bands located at <1750 and >1760 cm^−1^, as given in inset, are emphasized in blue and red, respectively. The area subtended by signals at >1760 cm^−1^ (cf. wavenumbers labeled in red) were used to compute the oxidation ratio, R_ox_, as discussed in Section 4.3.

As shown in the difference spectra of Figure 3, however, all studied stevia products, independent of their production procedure, were far from being homogeneous Reb M compounds. In agreement with previous Raman calibrations on pure rebaudioside compounds [17], calibration plots are available, through which we could retrieve two important structural parameters; one is linked to the total number of glucose rings on both C19 and C13 sides, N_tot_, and another one only to the number of rings on C19 side, N_19_. The calibration plots for N_tot_ and N_19_ are given in Figure 2c and Figure 2d, respectively. Figure 6a–c shows enlarged and deconvoluted Raman regions in the spectral interval 825~950 cm^−1^ for natural, fermented, and bioconverted samples, respectively (cf. labels in inset). From this spectral region, the spectroscopic areal ratio between bands at 887 and 898 cm^−1^ was computed to retrieve (from the plot in Figure 2c) the total number of rings, N_tot_, for all commercial products. In a similar way, from the deconvoluted bands in the wavenumber interval between 1700 and 1750 cm^−1^, given in Figure 7a, Figure 7b and Figure 7c for natural, fermented, and bioconverted samples, respectively (cf. labels in inset), one could compute (from the plot in Figure 2d) the number of rings on the C19 side, N_19_. Individual results of N_tot_ computations for different lots of commercial products are shown in Figure 6d, Figure 6e and Figure 6f for natural, fermented, and bioconverted samples, respectively. Similarly, in Figure 7d, Figure 7e and Figure 7f, individual N_19_ values are reported for different lots of natural, fermented, and bioconverted samples, respectively. From the N_tot_ and N_19_ parameters, we computed the number of glucose rings on the C13 side of the molecule, N_13_ = N_tot_–N_19_; then, from average N_13_, N_19_, and N_tot_ values over different lots of each product, an additional parameter was established, which is referred to as the balance ratio, R_B_ = (N_tot_–N_19_)/N_tot_ = N_13_/N_tot_. According to the notions given in the above points (a)~(d) [29], the higher the R_B_ ratio the better the taste performance of the commercial product.

**Figure 8:**
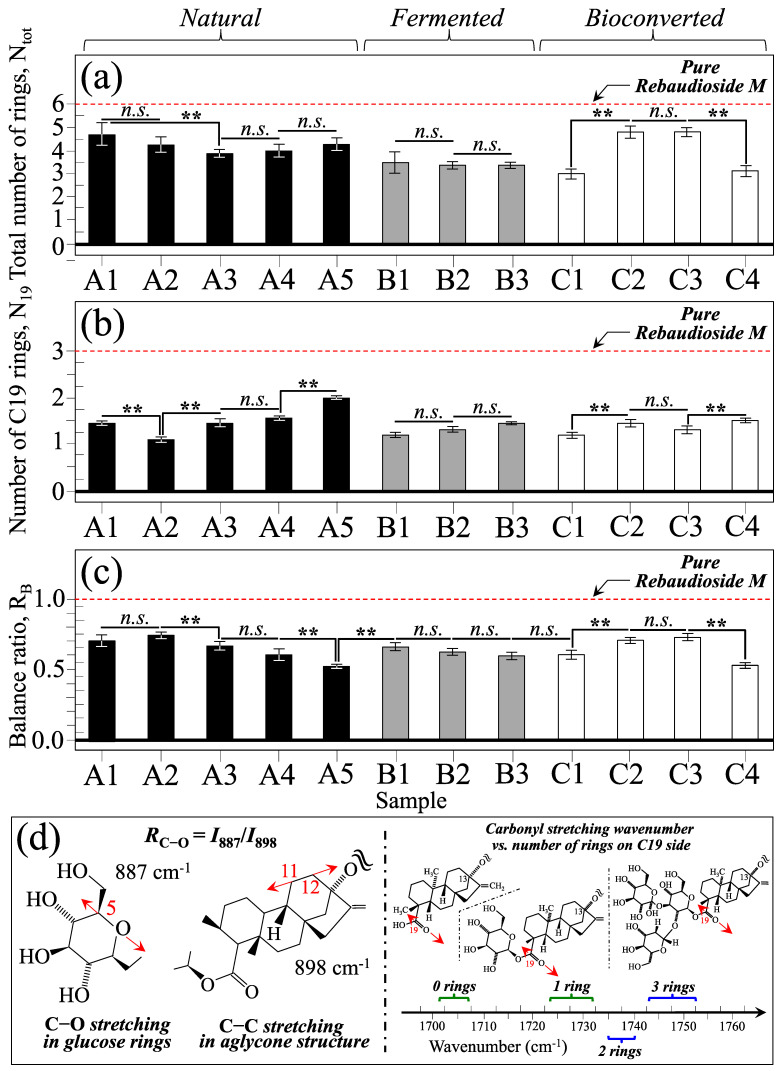
Assessment of ring structure and balance ratio. Plots of the parameters (**a**) N_tot_, (**b**) N_19_, and (**c**) R_B_ for all commercial products investigated. In (**d**), spectroscopic definitions are reported for the parameters N_tot_ and N_19_ [17]. Statistically non-significant differences were labeled as *n.s.*, while a value *p* < 0.05 was considered as statistically significant and labeled with two asterisks.

Figure 8a, Figure 8b and Figure 8c show plots of the parameters N_tot_, N_19_, and R_B_, respectively, as averaged over available lots for each of the commercial products investigated. In Figure 8d, spectroscopic definitions are schematically drawn for the parameters N_tot_ and N_19_ [17]. As seen, the sample A1 showed the highest N_tot_ value, but also two bioconverted products (C2 and C3) displayed a similar performance. This observation suggests that bioconversion is a promising approach, capable to produce products with a sensorial performance comparable to the best available natural products. All fermented products were instead characterized by relatively low N_tot_ values. Regarding the balance ratio, R_B_, two natural (A1 and A2) and two fermented (C2 and C3) were the products with the highest balance ratio, which positively impacts on their taste performance as compared with other products.

According to the above descriptions, one could state that the Raman spectroscopic information brings about a clear path to evaluate the sensorial performance, and thus the comparative quality, of the studied products. This point will be further discussed in the forthcoming Section 4.4.

### 4.3. Impact of Manufacturing Procedure on Non-Glycosidic Ester Impurities

Commercializing Reb M as a high-intensity sweetener requires strict control of the product chemical integrity, which is critical for both sensorial performance and purity control. This study shows that Raman spectroscopy can provide a rapid, non-destructive tool for assessing the compositional fidelity of Reb M products in comparison with selected spectral features from a reference high-purity sample. In the present analysis of commercially available Reb M products, a series of Raman bands above 1760 cm^−1^ were observed in the carbonyl region (cf. Figure 7) that cannot be attributed either to the glycosidic structure of high-purity Reb M (cf. Figure 2b) or to those of other rebaudiosides [17]. These features are consistent with the presence of (non-glycosidic) ester impurities belonging to the lactone family [40], likely corresponding to esterified diterpenoid byproducts or other carbonyl-rich non-Reb M species formed during extraction, purification, or downstream processing [41]. The detection of these lactone-type components unequivocally indicate the presence of process-induced impurities rather than aromatic or phenolic contaminants, highlighting the utility of Raman spectroscopy for differentiating steviol glycosides from closely related esterified species in commercial formulations. Lactones in Raman analysis of Reb M should thus be considered as representing steviol-derived degradation products or co-extracted terpenoid lactones, chemically distinct from phenolic contamination.

Although not widely reported, *stevia rebaudiana* might contain non-glycosidic terpenoids including possible lactones that could contribute to taste and physiological effects. The sesquiterpenoids in *Stevia rebaudiana* include various isomers, such as Sterebin I, J, E, F, M, and N (C15 terpenes), which could be precursors of lactone or related compounds with carbonyls [42]. These metabolites have been detected in both polar and non-polar extracts and reported to possibly contribute, although in minor extent, to the bitter aftertaste of Stevia products [42,43]. However, only sesquiterpenoids with cyclic ester (lactone), namely, sesquiterpene lactones, show Raman carbonyl bands at above 1750 cm^−1^. The sesquiterpene lactones with carbonyl stretching modes in the region 1750~1800 cm^−1^ are thus γ-lactones [40]. Spectral features in Figure 7 suggest that, under processing conditions and especially upon fermentation, relatively large amounts of oxidation products might form as new compounds [41]. According to the presence of carbonyl Raman bands at >1760 cm^−1^, we should consider lactones and cyclic esters as the most relevant impurities in the fermented products investigated. In other words, fermentation converts trace sesquiterpenoids and other terpenoid precursors already present in stevia into a family of oxygenated γ-lactones, including unsaturated, saturated, and strained variants, which likely coexist and give rise to multiple high-wavenumber C=O stretching Raman signals (cf. wavenumbers labeled in red in Figure 7).

**Figure 9:**
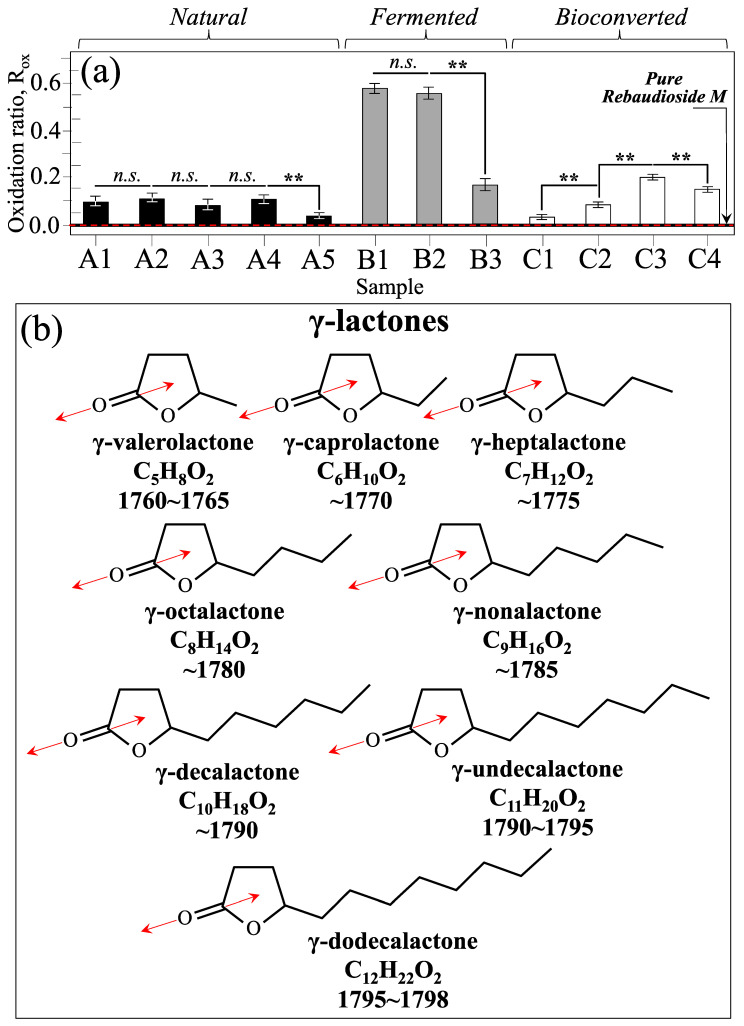
Assessment of the oxidation parameter. (**a**) Plot of R_ox_ for all tested commercial samples and (**b**) schematic drafts of different structures of γ-lactones and wavenumbers expected for their respective C=O stretching signals (cf. red arrows). Statistically non-significant difference were labeled as *n.s.*, while a value *p* < 0.05 was considered as statistically significant and labeled with two asterisks.

The relatively high intensity of γ-lactone-type signals in bacterially fermented Reb M products compared to plant-extracted or enzymatically bioconverted ones, represents the marker for a unique biochemical environment associated with microbial processing. During fermentation, enzymatic activities such as β-glucosidase-mediated deglycosylation generate free steviol or partially deglycosylated steviol-derived intermediates, exposing reactive hydroxy groups in close proximity to the C19 carboxy. Concurrently, fermentation typically proceeds under mildly acidic conditions due to the accumulation of organic acids, which provides favorable catalysis for intramolecular esterification reactions. In addition, bacterial oxidoreductase activity can introduce reposition of hydroxy groups on the diterpene scaffold, yielding γ-hydroxycarboxylic acid motifs that readily undergo cyclization. From a kinetic perspective, it is conceivable to consider that a five-membered ring closure is intrinsically favored over six-membered alternatives, and enzymatic pre-organization of intermediates further facilitates γ-lactone formation. As a result, γ-lactone-type non-glycosidic ester impurities can accumulate at low but detectable levels in fermented stevia products (Figure 9b), representing process-induced, steviol-related esterified byproducts rather than extraneous aromatic or phenolic contaminants.

We then computed a parameter referred to as the oxidation ratio, R_ox_, as the ratio between the cumulative areas of band components at >1760 cm^−1^ (cf. bands with wavenumbers labeled in red in Figure 7) and those comprised in the wavenumber interval 1700~1750 cm^−1^ (cf. bands with wavenumbers labeled in blue in Figure 7). A plot of R_ox_ for all tested commercial samples is given in Figure 9a, while Figure 9b schematically shows the different structures of γ-lactones and the wavenumbers expected for their respective C=O stretching signals.

Among the three bacterially fermented Reb M products examined here, two products (B1 and B2), showed the highest R_ox_ values (~0.6), while one (B3) exhibited a level only slightly higher but yet comparable to plant-extracted materials (cf. Figure 9a). This suggests that specific fermentation parameters can significantly influence lactone formation. The reduced γ-lactone content in B3 product is likely attributable to differences in microbial strain selection, as certain strains possess lower β-glucosidase and esterase activity toward Reb M, thereby limiting the formation of free steviol or partially deglycosylated intermediates that serve as precursors for γ-lactonization. In addition, controlled fermentation conditions, including maintenance of a neutral or mildly acidic pH, lower temperature, and shorter processing times, would reduce acid-catalyzed intramolecular esterification and minimize the kinetic favorability for five-membered ring closure. Redox management, such as limited oxygen exposure, may further suppress enzymatic or oxidative formation of γ-hydroxy acids, which are necessary intermediates for lactone cyclization. Finally, prompt downstream purification and stabilization likely prevent post-fermentation lactone formation. Collectively, these factors indicate that careful optimization of microbial strain, physicochemical conditions, and post-fermentation handling can yield fermented Reb M products with γ-lactone levels approaching those of high-purity plant extracts. From this perspective, the product labeled as B3 was by far the one produced with the most refined fermentation procedures.

As shown in Figure 9b, a semi-quantitative correlation can be drawn between the carbon chain length of γ-lactones and the position of their carbonyl Raman bands above 1750 cm^−1^. In general, a five-membered γ-lactone, such as γ-valerolactone, with its high ring strain, reduced hydrogen bonding, and shorter alkyl substituents, is expected to exhibit C=O stretching Raman signals near 1760–1765 cm^−1^, whereas lactones with progressively longer alkyl chains, including γ-caprolactone, γ-heptalactone, γ-octalactone, γ-nonalactone, γ-decalactone, γ-undecalactone, and γ-dodecalactone should show incremental increases in C=O stretching wavenumber up to ~1800 cm^−1^. This trend arises because the carbonyl stretching frequency is influenced by ring strain, substitution at the γ-position, and electron density along the alkyl chain, with longer chains slightly enhancing the carbonyl bond polarization and vibrational frequency. The higher ν_C=O_ wavenumber of five-membered lactones compared to six-membered lactones (e.g., δ-lactones) is likely due to an increased ring strain, which shortens and strengthens the C=O bond, raising its vibrational frequency; six-membered rings could be less strained and therefore exhibit slightly lower carbonyl wavenumbers. Although overlapping bands and matrix effects in complex extracts prevent precise peak-to-compound assignment, this semi-quantitative relationship may suffice in providing a useful rationale for interpreting the discrete series of high-wavenumber Raman bands observed in fermented stevia products as corresponding to γ-lactones of varying chain lengths.

Trace processing-derived constituents detected in commercial stevia products are generally expected to contribute measurably to the bitter and lingering aftertaste commonly associated with steviol glycoside sweeteners [42,43,44]. In particular, minor diterpenoid lactones and oxidized steviol fragments formed during extraction, purification, or thermal processing retain the hydrophobic steviol core while introducing carbonyl functionalities; a structural motif known to enhance activation of human bitter taste receptors (TAS2Rs) and to produce delayed, persistent bitterness even at very low concentrations. Oxidized carbohydrate excipients, such as cyclic esters (i.e., the γ-lactones reported here), maltodextrin-derived aldehydes, and carboxylic acids generally exhibit limited intrinsic bitterness, but could indirectly amplify bitterness perception by suppressing sweetness intensity and contributing metallic, astringent, or drying sensory notes that prolong aftertaste. Collectively, the bitter aftertaste of commercial stevia formulations is not solely an inherent property of rebaudioside molecules, but is significantly modulated by trace processing-derived impurities and oxidized excipients, consistent with the improved sensorial quality reported for higher-purity rebaudioside preparations and more tightly controlled manufacturing processes.

### 4.4. The Raman Quality Profile

As discussed in the previous sections, Raman spectroscopy can be exploited both to confirm the molecular identity of Reb M and to discriminate its production pathway through a chemically interpretable multivariate framework. A Raman Quality Profile (RQP) can then be constructed by integrating in a triangular plot three orthogonal spectral descriptors given in percents and based on the Raman parameters previously defined as: (i) heterogeneity and molecular crystallinity as indexes of structural homogeneity, (ii) glycosidic completeness and balance as indexes of sensorial performance, and (iii) biosynthetic effects linked to oxidative effects, as an index for the accuracy of production procedures.

A parameter representing the degree of structural homogeneity and molecular crystallinity, D_SH_ = (1-R_D_)R_cry_, was defined in relation to the spectral deviation index, R_D_, and the molecular crystallinity index, R_cry_, as described in Section 4.1. This parameter in percents provides sensitivity to residual polysaccharides and, more generally, to molecular disorder with respect to the pure Reb M molecule. In addition, a parameter referred to as the degree of sensorial performance, D_SP_, was defined as the sum of the total fraction of glucose rings (normalized with respect to a pure Reb M product), R_R_ = N_tot_/6, and the balance ratio, R_B_, as defined in Section 4.2. The parameter D_SP_, normalized with respect to the pure Reb M molecule, comprehensively gives a measure of glycosidic completeness and structural balance of the molecule, both these aspects impacting on the sensorial performance. Finally, an important aspect of product processing procedure related to the presence of non-glycosidic ester impurities as oxidation products was addressed by means of a degree of oxidation, D_O_, selected as coincident with the oxidation ratio, R_ox_, defined in Section 4.3. This parameter, normalized in percentage with respect to a value in which the areal fraction of carbonyls from oxidized impurities equals that of carbonyls from aglycone core, could be considered as a discriminant variable locating the level of manufacturing control.

**Figure 10:**
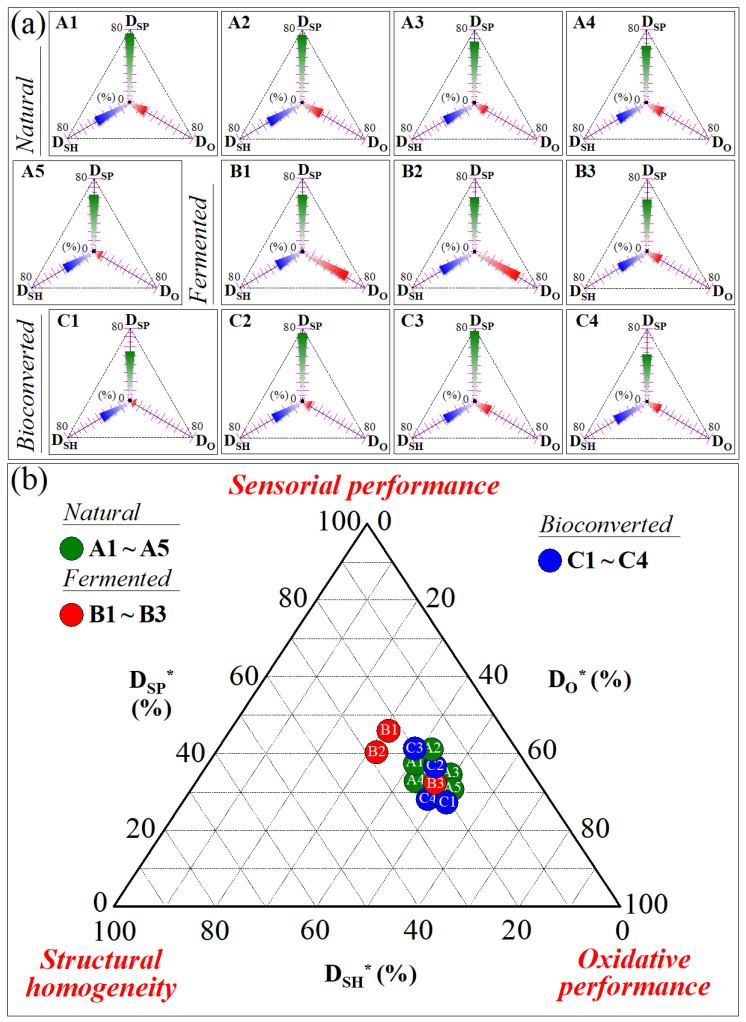
Sensorial, structural, and oxidative performances of commercial products. RQPs for all commercial products examined in this study; the plots in (**a**) condense three chemically meaningful dimensions into a scalar profile, enabling objective classification of Reb M products according to production pathways rather than nominal purity alone. A degree of structural homogeneity, also including molecular crystallinity, D_SH_ = (1 − R_D_)R_cry_, is defined in relation to the spectral deviation index, R_D_, and the molecular crystallinity index, R_cry_, as described in Section 4.1; the parameter referred to as the degree of sensorial performance, D_SP_, was defined as the sum of the total fraction of glucose rings (normalized in percents with respect to a pure Reb M product), R_R_ = N_tot_/6, and the balance ratio, R_B_, as defined in Section 4.2; and, the percent degree of oxidation, D_O_, was selected as coincident with the oxidation ratio, R_ox_, defined in Section 4.3. In (**b**), a triangular plot is given of normalized percent parameters describing the overall performance within each commercial product; sensorial performance, D_SP_*, structural homogeneity, D_SH_*, and oxidative performance, D_O_* = 1 − D_O_, are the three employed parameters whose asterisk indicates normalization within the overall characteristics of each product. Natural (A1~A5), fermented (B1~B3), and bioconverted samples (C1~C4) in the plot in (**b**) are displayed in green, red, and blue, respectively (cf. sample names in inset to each data plot).

The resulting RQPs for all commercial products examined are shown in Figure 10a. As mentioned above, these plots condense three chemically meaningful dimensions into a scalar profile, enabling objective classification of Reb M products according to production pathways rather than nominal purity alone. Interestingly, all commercial products, independent of the manufacturing procedure, experienced a relatively low degree of structural homogeneity, D_SH_, comprised in a relatively narrow interval 35~41%. On the other hand, only two natural products experienced a high sensorial performance (D_SP_ = 73~75% for A1 and A2), while all natural products encompassed relatively low degrees of oxidation (D_O_ < 22%). This was not always the case for fermented products, two of which (B1 and B2) showed much higher degrees of oxidation (D_O_ = 58 and 56%, respectively). An interesting result was that two bioconverted products (C2 and C3), while showing quite high degrees of sensorial performance, comparable with those of the best natural products, also met quite low oxidation characteristics. In other words, the present data confirmed the superior quality of natural products but also showed the power of bioconversion as an advanced manufacturing process capable of producing stevia products with characteristics comparable with those of natural products.

A final plot is given in Figure 10b, in which we have normalized in percents the overall performance within each commercial product in a triangular plot comprising sensorial performance, D_SP_*, structural homogeneity, D_SH_*, and oxidative performance, D_O_*, where the asterisk is added to D_SP_, D_SH_, and D_O_ merely to indicate normalization of these parameters within the characteristics of each product. Natural products with a good balance among the above three characteristics can be spotted in A1 and A2, while two fermented products, B1 and B2, were skewed towards poor oxidative performance and clearly need processing improvements. Among bioconverted products, C3 showed very good sensorial performance and structural homogeneity, while there are calls for efforts in improving its oxidative performance. An inverse pattern is found for bioconverted C1, which had the best oxidative among all the studied products, but yet requires improvement in its sensorial performance.

In conclusion, the RQP plot helps to locate the strong and weak points of each commercial product, and could help end users to evaluate quality and manufacturers to improve them.

### 4.5. The Importance of Raman Spectroscopy in Assessing Stevia Sweeteners

Looking at the published literature, one could locate X-ray diffraction (XRD) and differential scanning calorimetry (DSC) as the classical techniques for crystallinity assessments in food powders [45,46,47]. However, the application of these analytical methods to commercial stevia powders is hardly straightforward. Published XRD/DSC studies on stevia-derived materials have mainly focused on highly purified individual steviol glycosides and isolated polymorphs such as rebaudioside D [45] or crystalline stevioside solvates [46]. In contrast, commercially formulated powders are composed of mixtures of steviol glycosides and additional excipients, exhibiting partial amorphization, hydration, and polymorphic heterogeneity. Under these conditions, XRD patterns are typically broadened and difficult to quantitatively interpret, while DSC thermal events may overlap because of water loss, polymorphic transitions, and decomposition phenomena.

Raman spectroscopic analyses, as developed here, enable direct molecular-level probing of local structural order without sample pretreatment and can detect subtle variations in intermolecular organization even in heterogeneous powder mixtures. Moreover, Raman analysis has already been demonstrated as an effective tool for stevia product characterization and quality control [17,48]. We therefore consider Raman spectroscopy not merely complementary to XRD/DSC, but particularly advantageous for the analysis of complex commercial stevia formulations where conventional crystallinity measurements may not provide unambiguous quantitative information. In addition, commercial stevia products might contain minor fractions of maltodextrin, erythritol, or other carriers/adulterants, which could alter XRD patterns and DSC thermograms [48]. In contrast, Raman spectroscopy appears to be uniquely capable of providing unambiguous quantitative information for heterogeneous stevia formulations and, unlike XRD, to allow the establishment of molecular-order parameters more suitable for standardization.

## 5. Conclusions

In conclusion, this study demonstrates that Raman spectroscopy represents a robust, rapid, and economically sustainable platform for the authentication and functional assessment of Reb M across different manufacturing routes. By establishing three quantitative spectroscopic parameters, which address compositional homogeneity, predicted sensorial performance, and oxidative stability, we move beyond simple origin discrimination and introduce a multidimensional quality framework. All commercial products, irrespective of whether derived from natural leaf extraction, microbial fermentation, or enzymatic bioconversion, exhibited measurable deviations from highly pure Reb M, revealing a non-negligible degree of structural and compositional inhomogeneity. Nevertheless, important process-dependent trends emerged. While certain naturally derived products achieved a favorable balance between sensorial and oxidative performance despite inter-brand variability, a significant fraction of fermentation-derived samples displayed inferior oxidative stability, suggesting process-related residuals or matrix imbalances that may affect long-term quality. In contrast, enzymatic bioconversion appeared as a particularly promising technological strategy, yielding products characterized by superior sensorial profiles and minimal oxidative signatures.

Collectively, these findings underscore that Raman spectroscopy is not merely an authentication tool, but a comprehensive quality-assurance methodology capable of correlating molecular structure with functional performance. Its near-zero marginal cost, rapid implementation, and non-destructive nature position it as a strategic alternative to conventional chromatographic approaches, enabling real-time monitoring and promoting transparency, safety, and consistency in the evolving landscape of next-generation steviol glycoside sweeteners. Future study should focus on the standardization of Raman technology for both on-line quality control and clarity towards potential customers.

## Data Availability

The original contributions presented in this study are included in the article/Supplementary Materials. Further inquiries can be directed to the corresponding author.

## Supplementary Materials

The following supporting information can be downloaded at: https://www.mdpi.com/article/10.3390/foods15111994/s1, Figure S1: Results of PCA analysis on the Raman spectra of all batches available for different commercially available stevia products: natural (A), microbially fermented (B), and enzymatically bioconverted products (C). The plot envisages the highest degree of inhomogeneity across batches of microbially fermented samples.
