# Supplementary material for: Raman Spectroscopic Authentication of Rebaudioside M: Discriminating Natural, Fermentation-Derived, and Enzymatically Bioconverted Stevia Sweeteners
Source: Foods. 2026 Jun 3;15(11):1994. doi: 10.3390/foods15111994 (PMC13257270; doi:10.3390/foods15111994)
Supplement: Supplementary file 1 [file foods-15-01994-s001.zip › foods-4316496-supplementary.pdf]

Supplementary Information

Principal component analysis (PCA) was performed using a custom Python script (Python 3.13.9, Anaconda distribution). The spectral dataset (CSV format) was imported and processed using Pandas 2.3.3. Prior to PCA, the spectral matrix was mean-centered without variance scaling using the *StandardScaler* function (from scikit-learn 1.7.2. PCA was conducted using the *PCA* module of scikit-learn, and the first two principal components (PC1 and PC2) were extracted. Numerical computations, including eigenvalue decomposition and loading vector calculations, were performed using NumPy 2.3.5. All visualizations (score plots, loading plots, and peak annotations) were generated using Matplotlib (bundled with the Anaconda Python 3.13.9 environment). Ranking the absolute loading values identified the top contributing wavenumbers for each principal component. The PCA analysis on the spectra was applied over the wavenumber interval 200~2000 cm^{-1} for all available batches of natural (A), microbially fermented (B), and enzymatically bioconverted products (C) (cf. also Section 2.1 of the main text).

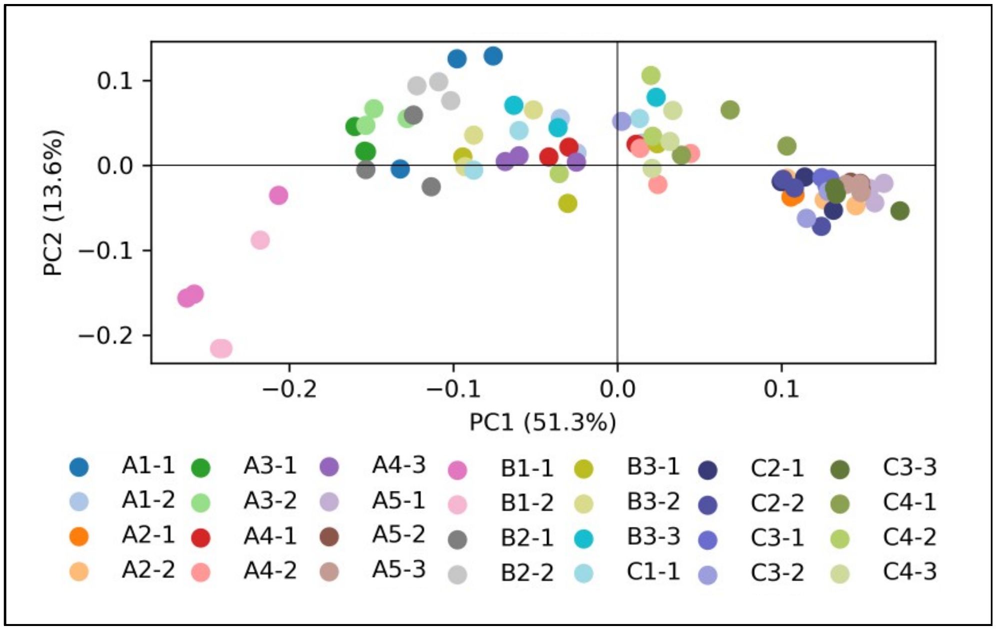

Figure S1: Results of PCA analysis on the Raman spectra of all batches available for different commercially available stevia products: natural (A), microbially fermented (B), and enzymatically bioconverted products (C). The plot envisages the highest degree of inhomogeneity across batches of microbially fermented samples.
